# Cardiac small-conductance calcium-activated potassium channels in health and disease

**DOI:** 10.1007/s00424-021-02535-0

**Published:** 2021-02-23

**Authors:** Xiao-Dong Zhang, Phung N. Thai, Deborah K. Lieu, Nipavan Chiamvimonvat

**Affiliations:** 1 Division of Cardiovascular Medicine, Department of Internal Medicine, School of Medicine, University of California, Davis, One Shields Avenue, GBSF 6315, Davis, CA 95616 USA; 2 Department of Veterans Affairs, Northern California Health Care System, 10535 Hospital Way, Mather, CA 95655 USA; 3 Department of Pharmacology, School of Medicine, University of California, Davis, Davis, CA 95616 USA

**Keywords:** Small-conductance Ca^2+^-activated K^+^ channel, SK channel, Calcium, Cardiac action potential, Cardiac repolarization, Atrial fibrillation, Cardiac arrhythmia, Apamin, Heart failure, Calmodulin, Pulmonary vein, Pacemaking cell

## Abstract

Small-conductance Ca^2+^-activated K^+^ (SK, K_Ca_2) channels are encoded by *KCNN* genes, including *KCNN*1, 2, and 3. The channels play critical roles in the regulation of cardiac excitability and are gated solely by beat-to-beat changes in intracellular Ca^2+^. The family of SK channels consists of three members with differential sensitivity to apamin. All three isoforms are expressed in human hearts. Studies over the past two decades have provided evidence to substantiate the pivotal roles of SK channels, not only in healthy heart but also with diseases including atrial fibrillation (AF), ventricular arrhythmia, and heart failure (HF). SK channels are prominently expressed in atrial myocytes and pacemaking cells, compared to ventricular cells. However, the channels are significantly upregulated in ventricular myocytes in HF and pulmonary veins in AF models. Interests in cardiac SK channels are further fueled by recent studies suggesting the possible roles of SK channels in human AF. Therefore, SK channel may represent a novel therapeutic target for atrial arrhythmias. Furthermore, SK channel function is significantly altered by human calmodulin (CaM) mutations, linked to life-threatening *arrhythmia* syndromes. The current review will summarize recent progress in our understanding of cardiac SK channels and the roles of SK channels in the heart in health and disease.

## Introduction

Ca^2+^ ions are the central player in the regulation of cardiac excitability and contractility. Ca^2+^ influx through voltage-gated Ca^2+^ channels is critical not only for initiating cardiac excitation-contraction coupling, but also for activating multiple downstream signaling pathways in response to changes in membrane potentials. Small-conductance Ca^2+^-activated K^+^ (SK, K_Ca_2) channels, encoded by *KCNN* genes, are among the Ca^2+^-gated ion channels that play critical roles in the regulation of cardiac excitability. The dynamic beat-to-beat changes of intracellular Ca^2+^ are essential for the function and regulation of SK channels.

Our understandings of cardiac SK channels have greatly expanded in the past 2 decades since the functional and molecular identification of SK channels in the heart [99]. Ca^2+^-activated K^+^ currents (*I*_*K,Ca*_) in the heart have previously been considered controversial [26, 69]. However, studies by our group and others have provided evidence to substantiate the pivotal roles of SK channels, not only in healthy heart but also with diseases including atrial fibrillation (AF), ventricular arrhythmia, and heart failure (HF) [10, 27, 28, 34, 59, 71, 99, 107]. SK channels are prominently expressed in atrial myocytes and pacemaking cells, compared to ventricular cells [13, 92, 93, 99, 105]. However, the channels are significantly upregulated in ventricular myocytes in HF and pulmonary veins (PV) in AF models. The potential role of SK channels in human AF has been demonstrated using genome-wide association analysis (GWAS), revealing an association between single-nucleotide polymorphism (SNP) in *KCNN2 *and* KCNN3* genes with lone AF [27, 28]. SK channels may represent novel therapeutic targets against AF [10, 19–21, 76, 82, 84, 88, 95]. Furthermore, SK channel function is significantly altered by human calmodulin (CaM) mutations, linked to life-threatening *arrhythmia* syndromes [38, 81, 104]. The current review will summarize recent progress in our understanding of cardiac SK channels and serve as a discussion platform for the roles of SK channels in the heart in health and disease.

## SK channels

The discovery of SK channels dates back to more than 70 years ago [107]. The bee venom toxin apamin, a highly selective blocker of SK channels, enables the molecular identification of SK channels in the mammalian brain [47], which were later observed in various tissues, including smooth muscle, endothelia, epithelia, blood cell, and heart [85, 107]. The family of SK channels consists of three members with differential sensitivity to apamin: SK1 (or K_Ca_2.1 encoded by *KCNN1* gene), SK2 (or K_Ca_2.2 encoded by *KCNN2* gene), and SK3 (or K_Ca_2.3 encoded by *KCNN3* gene) [1, 107]. SK2 is highly sensitive to apamin, with a half-blocking concentration (EC_50_) of ~40 pM, whereas SK1 channels are the least sensitive to apamin with an EC_50_ of ~10 nM. SK3 channels show intermediate sensitivity, with an EC_50_ of ~1 nM [1]. No other class of K^+^ channels is blocked by apamin. An intermediate-conductance Ca^2+^-activated K^+^ channel (IK, SK4, or K_Ca_2.4 encoded by the *KCNN4* gene) is structurally and functionally similar to the SK channels and is classified in the same gene family [1, 85]. Functional SK channels assemble to form homomeric or heteromeric tetramers [47, 87, 94]. A recent cryo-electron microscopy (EM) study reveals the structure of human SK4-CaM channels in closed and activated states [48]. It was found that one channel tetramer binds to four CaM molecules. The C-lobe of CaM binds to the channel constitutively, whereas the N-lobe of CaM interacts with the S4-S5 linker in a Ca^2+^-dependent manner. The S4-S5 linker undergoes conformational changes upon CaM binding to open the channel pore [48]. This structural study lays essential foundations in revealing the physiological and pharmacological properties of SK channels in the heart.

## Functional expression of SK channels in the heart

All three isoforms of SK channels are expressed in mouse and human hearts [93, 99]. The channels are expressed in different regions of the hearts in multiple species, including rabbit PV [13, 75] and ventricular myocytes [16, 49], human atrial myocytes [83, 103], and rat ventricular myocytes [72]. SK currents have also been recorded in canine PV and left atrial myocytes using an SK-specific blocker, NS8593 [77]. In addition, SK channels play critical roles in pacemaking cells, including mouse atrioventricular nodal cells [105] and rabbit and mouse sinoatrial nodal cells [13, 92]. SK channels are more abundantly expressed in atrial myocytes, pacemaking cells, and cardiac Purkinje fibers, compared to ventricular myocytes [79, 92, 99, 105]. SK2 and SK3 channels are also functionally expressed in mouse embryonic cardiomyocytes and are required for maintaining excitability in developing cardiomyocytes, important in promoting Ca^2+^-dependent gene expression [44].

## Regulation of cardiac SK channels

### Cardiac SK channel interactome

Similar to other membrane proteins, ion channels form multi-protein complexes interacting with the extracellular matrix and cytosolic proteins [2, 9, 63]. SK channels are gated solely by beat-to-beat changes in intracellular Ca^2+^ *via* CaM, a ubiquitous Ca^2+^ sensing protein. CaM binds to the CaM binding domain (CaMBD) in the C termini of SK channels. SK channels are activated upon Ca^2+^-dependent CaM binding to the S4-S5 linker of the SK channels, resulting in conformational changes leading to channel activation [1, 48, 98]. CaM is essential for Ca^2+^ sensitivity and critical to the trafficking of SK channels [62]. Specifically, previous studies have demonstrated that Ca^2+^-independent association between CaM and SK channels is necessary for cell surface expression.

Several cytoskeletal proteins including α-actinin2 [55, 56], filamin A [78], and myosin light chain 2 (MLC2) [57] interact with cardiac SK2 channels. Specifically, α-actinin2 and MLC2 interact *via* the C-termini, and filamin A interacts *via* the N-termini of the SK2 channel. Moreover, cardiac SK2 channels couple with L-type Ca^2+^ channels, Ca_v_1.3 and Ca_v_1.2, through a physical bridge, α-actinin2. Trafficking of SK2 channels is critically dependent on the direct protein-protein interactions of the channels with α-actinin2, MLC2, and filamin A [56, 57, 78]. Knockdown of α-actinin2 or filamin A results in a decrease in SK2 channel expression on the membrane and localization of SK2 channels in the endosome, suggesting a reduction in the recycling of SK2 channels from the endosome. Finally, SK2 channel trafficking is Ca^2+^-dependent in the presence of α-actinin2. A decrease in intracellular Ca^2+^ results in a significant reduction of SK2 channel membrane localization [78]. Therefore, an increase in intracellular Ca^2+^, as evident during rapid AF or atrial tachycardia, is predicted to increase SK2 channel expression, leading to shortening of the atrial APs and maintenance of arrhythmias. One previous study using rapid pacing in isolated rabbit atria demonstrates a significant increase in SK2 immunostaining from a perinuclear pattern to the plasma membrane in the PV after burst pacing, suggesting an increase in forward trafficking of SK2 channels [75]. Indeed, PV is known to be the critical initiation site of human AF [35].

Protein kinase (casein kinase II, CK2) and protein phosphatase 2A (PP2A) are identified as SK2 channel binding proteins [3, 6]. CK2 and PP2A regulate SK channel sensitivity to intracellular Ca^2+^ by phosphorylating or dephosphorylating CaM [1]. In the heart, CK2 is found to co-localize with SK2 and SK3 in the rat ventricle. The increased expression of PP2A and the decreased interaction of CK2 and SK2 are two mechanisms underlying the upregulation of cardiac SK currents in the rat model of volume-overload HF [100]. A recent study found that junctophilin type 2 (JP2) associates with SK2 channels in cardiomyocytes [29]. JP2 directly interacts with SK2 channels *via* the membrane occupation and recognition nexus (MORN motifs) in its N-terminus.

**Fig. 1:**
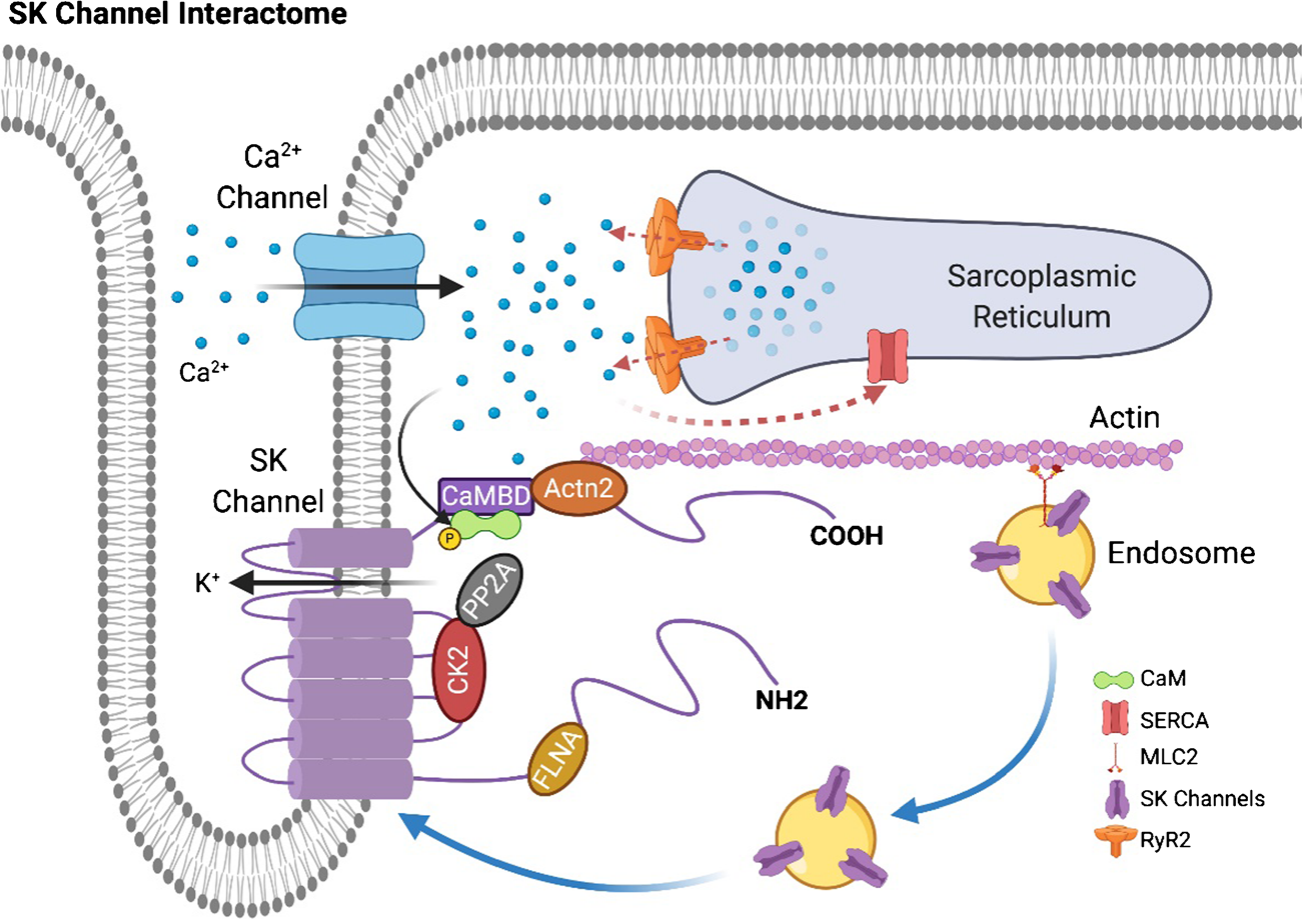
SK channels interactome. SK channels interactome includes α-actinin2 (Actin2), filamin A (FLNA), myosin light chain 2 (MLC2), CK2, and PP2A. Cardiac SK channels have been shown to couple to L-type Ca^2+^ channels through a physical bridge, α-actinin2. SK2 channels do not physically interact with the Ca^2+^ channels, instead the two channels co-localize via their interaction with α-actinin2 along the Z-line in atrial myocytes. An increase in intracellular Ca^2+^, as evident during rapid AF or atrial tachycardia, is predicted to increase SK2 channel expression leading to shortening of the atrial APs and maintenance of arrhythmias. Schematic representation was generated using BioRender

In summary, the SK channel interactome in cardiomyocytes consists of homomeric or heteromeric SK channel α subunits, CaM, α-actinin2, filamin A, CK2, and PP2A (Fig. 1). The interactions of the multi-protein SK channel complexes are critical for the expression, function, and trafficking of SK channels in cardiomyocytes. Additional interacting proteins of SK channels are likely to be identified with advanced proteomics approaches.

### Coupling of SK channels with L-type Ca^2+^ channels and ryanodine receptors

**Fig. 2:**
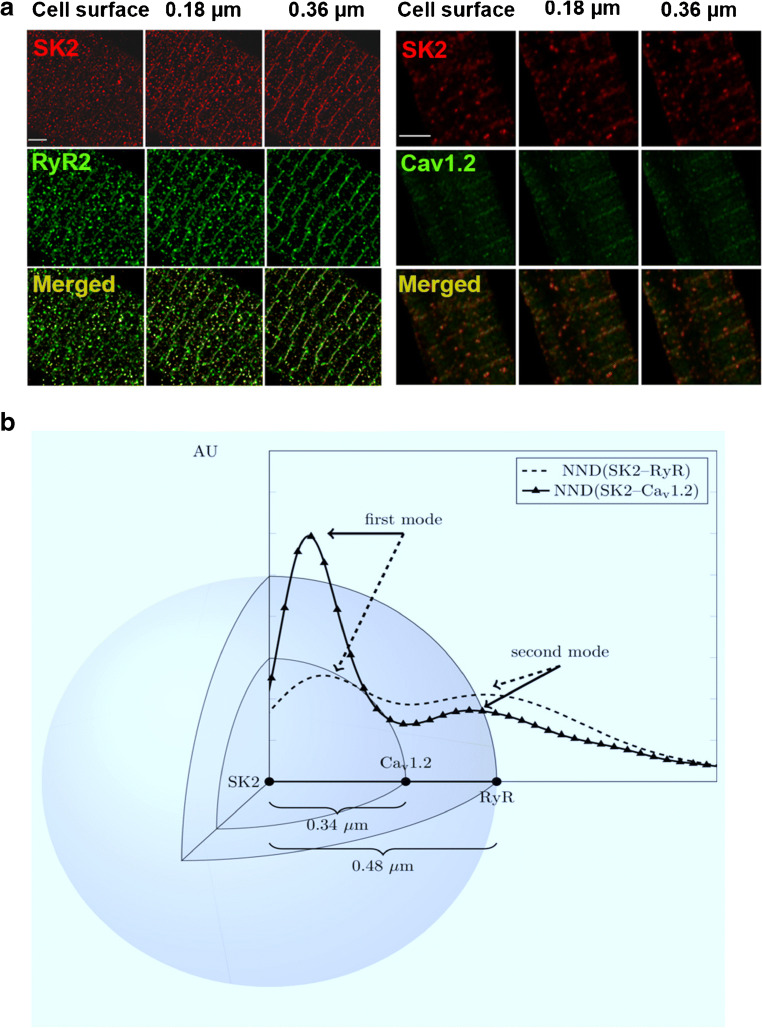
Spatial distribution of SK2, RyR2, and Ca_v_1.2 [108]. **a** Spatial coupling of SK2, RyR2, and Ca_v_1.2 channels. Stimulated emission depletion (STED) microscopy of SK2, RyR2, and Ca_v_1.2 expression in rabbit ventricular myocytes at three Z planes (scale bar 2 μm). **b** Visualization of the spatial distribution of SK2, RyR2, and Ca_v_1.2. (AU, arbitrary units; NND, nearest neighbor distance)

The co-localization of SK and Ca^2+^ channels suggests the possibility that local subsarcolemmal Ca^2+^, resulting from Ca^2+^ channel opening, is sufficient to activate SK channels, as was demonstrated for hippocampal neurons [61]. However, two previous studies suggest that sarcoplasmic reticulum (SR) Ca^2+^ release *via* ryanodine receptors (RyR2) is required for the activation of cardiac SK channels [66, 89]. Our recent report shows that L-type Ca^2+^ channels (LTCCs) provide the immediate Ca^2+^ microdomain for the activation of SK channels in cardiomyocytes, supporting the roles of both Ca^2+^ entry and Ca^2+^ release from SR in the activation of SK channels. Further super-resolution imaging quantifies and localizes the three molecules including SK channels, LTCCs, and RyR2 to be within hundreds of nanometers (Fig. 2). The distribution of nearest neighbor distances (NND) between SK2 and RyR2 as well as SK2 and Ca_v_1.2 was bimodal, suggesting a spatial relationship between the channels. Therefore, Ca^2+^ influx through LTCCs and Ca^2+^ release *via* RyR2 provide the immediate and efficient Ca^2+^ microdomain for the activation of SK channels [108].

### Phosphorylation of cardiac SK channels

Even though SK channels are expressed at a relatively low level in normal ventricles, SK channels have been shown to be phosphorylated by both Ca^2+^/CaM-dependent protein kinase II (CaMKII) and protein kinase A (PKA) in different models of cardiac hypertrophy, leading to the upregulation of SK currents in ventricular myocytes [37, 64]. Cardiac SK2 channels form multiprotein complexes with phosphorylated CaMKII at threonine-286 in a spontaneously hypertensive rat model. The modulation of the channels by CaMKII may contribute to ventricular arrhythmias during hypoxia in hypertrophied hearts [88]. Additionally, β-adrenergic stimulation results in enhanced SK channel activation *via* increased CaMKII activities [43]. In contrast, PKA phosphorylation at serine-465 of SK2 channel attenuates rectification of SK currents by reducing the voltage-dependent inhibition by intracellular Ca^2+^, leading to the upregulation of SK currents in ventricular myocytes from hypertrophic hearts [37]. In human AF, autophosphorylated CaMKII at Thr287 is significantly increased, leading to the upregulated SK currents in atria with increased Ca^2+^ sensitivity but decreased expression of SK1, SK2, and SK3 channels [30].

### MiRNA regulation of cardiac SK channels

MicroRNAs (miRNAs) are endogenously expressed small non-coding RNAs. MiRNAs in the heart have been extensively studied in the past decade [17, 31, 39, 73, 91, 109]. Cardiac-specific miRNAs play critical roles in heart development, myocardial regeneration, and cardiac remodeling. Recent studies have provided strong evidence for the critical roles of miRNAs in the regulation of cardiac ion channels and transporters [5, 7, 22, 25, 65, 90]. We have investigated the relationship between cardiac-specific *miRNA-1/133a*, cAMP signaling, and electrical remodeling in a clinically relevant mouse myocardial infarction (MI) model [68]. Chronic over-expression of inducible cAMP early repressor (ICER) from excessive β-adrenergic signaling in MI results in suppression of *miRNA-1 *and* miRNA-133a* expression leading to the reduction of transient outward K^+^ currents (*I*_to_) and prolonged action potential (AP) duration (APD) [68]. MiRNA 499 (MiR-499) is upregulated in the atria of patients with AF, leading to the downregulation of SK3 channels by binding to the 3' untranslated region of *KCNN3* gene [53]. The role of miRNAs in cardiac SK channels regulation requires further studies, especially in diseased hearts.

### Sex-specific regulation of cardiac SK channels

Expressions of SK2 channel and SK current density are higher in ventricular myocytes of female rabbits compared to male rabbits [14]. Furthermore, action potential triangulation, a known proarrhythmic predictor, is observed in female rabbit hearts upon isoproterenol stimulation, which could be reversed by apamin, suggesting the possible roles of SK channels in ventricular fibrillation in female animals during sympathetic stimulation. This report of sex-specific expression and function of cardiac SK channels highlights the critical need for a deeper understanding of sex-specific therapeutics in cardiac arrhythmia, an area that is currently understudied. Further investigations are necessary to determine the underlying mechanisms of the sex-specific expression of SK channels [23].

## Physiological significance of cardiac SK channels

### Critical roles in cardiac repolarization

Repolarization of cardiac AP is critically dependent on the orchestrated activity of multiple K^+^ channels and transporters. The initial study found that inhibition of SK currents by apamin prolonged APD in mouse and human atrial myocytes. However, the effects were less prominent in ventricular myocytes suggesting the unique role of the channels in atrial repolarization [99]. These findings are supported by subsequent studies in global *SK2* knockout mice, demonstrating that ablation of *KCNN2* results in a significant prolongation of APD, prominently in the late phase of the repolarization in atrial myocytes, and increased susceptibility to AF [51]. In contrast, there is no significant alteration in the APD in ventricular myocytes, and no ventricular arrhythmias are induced in the null mutant animals [51]. Conversely, gain-of-function of SK3 channels results in a significant shortening of APD in atrial myocytes [106].

Consistently, optical mapping in isolated canine left atria demonstrates that inhibition of SK channels by either apamin or UCL1684 [80] prolongs APD [41]. SK channel inhibitors, NS8593, and ICAGEN [32] prolong APD in isolated human atrial myocytes [83]. The above studies support the critical role of SK channels in the repolarization not only in mouse and canine but also in human atrial myocytes [71, 99]. In atrioventricular nodal cells, SK2 channel overexpression results in shortening of spontaneous APs and an increase in the firing frequency, while ablation of SK2 channels results in the opposite effects [105]. These findings are further supported by experimental data in rabbit heart [13], where sinoatrial node (SAN) cells exhibit larger SK currents than pulmonary vein cardiomyocytes. Furthermore, apamin decreases the firing rate and prolongs APD in SAN cells and pulmonary vein cardiomyocytes.

### Intracellular signaling and mitochondrial function

K^+^ channels are critical to normal mitochondrial function by regulating the mitochondrial membrane potentials, plasticity, matrix volume, reactive oxygen species (ROS) production, and respiratory chain activities [12, 97]. The activation of the mitochondrial K^+^ channels results in cardiac protective effects against ischemia-reperfusion (I/R) injury [54, 74]. Studies demonstrate that SK channels are expressed in neuronal and cardiac inner mitochondrial membrane [24, 86, 101]. An SK channel opener, DCEBIO, is found to protect the heart from the I/R injury, while an SK channel inhibitor, NS8593, antagonizes the protection by DCEBIO [86]. This is further supported by a study identifying the expression and functional effects of SK3 variants in the inner mitochondrial membrane of guinea pig, rat, and human ventricular myocytes [101], and *KCNN3* gene silencing with siRNA enhances cell death. The function of mitochondrial SK channels in Ca^2+^-dependent ventricular arrhythmia was further investigated in a rat model of cardiac hypertrophy [45]. A membrane-permeable SK enhancer, NS309, improves aberrant Ca^2+^ homeostasis, reduces the oxidation of ryanodine receptors, and abolishes ventricular tachycardia and fibrillation induced by β-adrenergic stimulation. Ca^2+^ signaling in mitochondria is critical to cardiac energetics, excitation-contraction (E-C) coupling, and cytosolic Ca^2+^ signaling [18]. Mitochondrial SK channels sense the mitochondrial Ca^2+^ signaling to regulate the mitochondrial function. Further studies are necessary to understand the molecular and cellular mechanisms of SK channels in the regulation of mitochondrial function in physiological and pathological conditions.

## Remodeling of cardiac SK channels in disease conditions

**Fig. 3:**
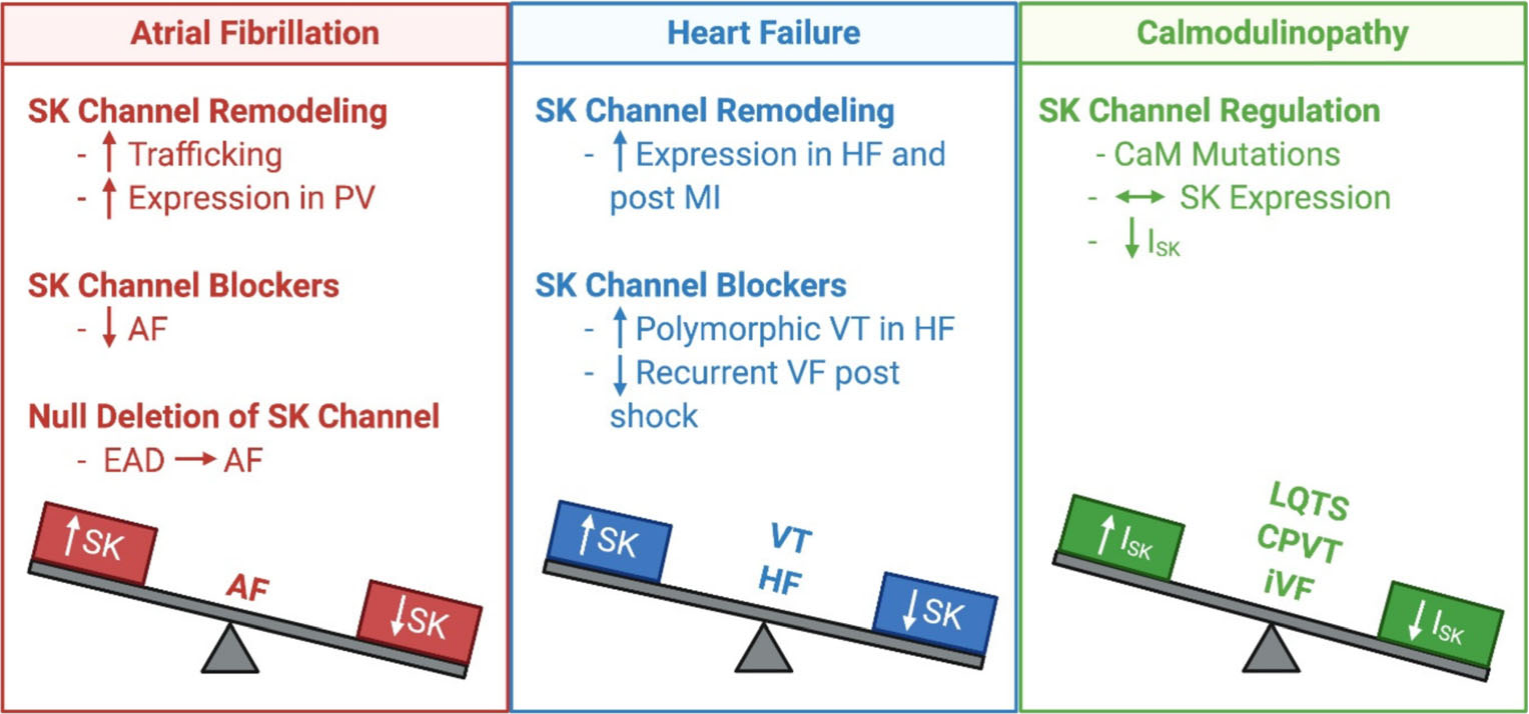
Functional roles of SK channels in normal and diseased hearts. Distinct roles of SK channels in atria and ventricles are depicted together with remodeling in AF, HF, and calmodulinopathy. EAD, early afterdepolarization; PV, pulmonary veins; AF, atrial fibrillation; VT/VF, ventricular tachycardia and fibrillation; LQTS, long QT syndromes; CPVT, polymorphic ventricular tachycardia; IVF, familial idiopathic ventricular fibrillation

Cardiac SK channel expression and function are significantly altered in disease conditions, as summarized in Fig. 3.

### Atrial arrhythmia

Emerging evidence from our group and others have shown that SK channels significantly contribute to AF. Ozgen *et al.* elegantly show that atrial burst pacing in rabbits, a model that mimicked ectopic foci, upregulates SK2 channel proteins in the cytosolic and membrane regions and shortened APD in PV [75]. Similarly, SK current is more prominently expressed in canine PV than left atrium (LA) and is upregulated by atrial tachypacing [77]. Mechanistically, this may result from Ca^2+^-dependent trafficking of SK2 channels to the membrane during rapid pacing [78].

The importance of SK channels in AF is further highlighted in a genome-wide association study that identified genetic variants in *KCNN2* and *KCNN3* genes that encode SK2 and SK3 channels be linked to AF susceptibility [27]. Overexpression of SK3 channels in mice results in increased sudden death due to AV block and severe sinus bradyarrhythmias [60]. In addition, atrial cardiomyocytes isolated from SK3 overexpressed mice exhibit significantly shortened APD with abbreviated atrial effective refractory period and increased AF inducibility [106]. In contrast, ablation of *KCNN2* results in a significant prolongation of APD and increased AF susceptibility [51]. These studies highlight that both gain- and loss-of-function of SK channels can increase AF susceptibility, likely from distinct underlying mechanisms. K^+^ channel gain-of-function is predicted to result in decreased atrial refractoriness and reentry mechanisms. On the other hand, K^+^ channel loss-of-function results in delayed repolarization and possibly triggered arrhythmias.

Downregulation of SK channel expression and current has also been documented in AF patients. Specifically, SK1, SK2, and SK3 channel expression and apamin-sensitive currents are reduced in chronic AF patients [83, 103]. Although the above studies highlight the crucial role for SK channel remodeling in AF, the exact timing for the observed changes in expression and function of SK channels in AF remains incompletely understood.

### Heart failure

Abnormal Ca^2+^ handling [58], complex alterations in gene expression [4], and posttranslational modification [50] have been documented in HF. SK channels are significantly upregulated in HF even though most other K^+^ channels are downregulated [10]. In a tachycardia-induced rabbit HF model, shortening of APD in ventricular myocytes and recurrent spontaneous ventricular tachyarrhythmias can be reversed by apamin due to an increase in apamin-sensitive K^+^ current (*I*_KAS_) [15]. Moreover, the apamin-sensitive K^+^ currents are heterogeneously upregulated, with higher density in the epicardial than mid myocardial and endomyocardial myocytes. The upregulation of *I*_KAS_ is due, in part, to increased Ca^2+^ sensitivity of the SK channels in HF. Importantly, *I*_KAS_ is also upregulated in failing human ventricles, attributed to an upregulation in SK2 expression and increased Ca^2+^ sensitivity [8]. Similar findings are reported in a canine right ventricular tachypacing model [8]. Indeed, SK channels’ upregulation in ventricular myocytes in HF may play a protective role by increasing repolarization reserve [40]. Moreover, SK channels are upregulated in atrial tissues from human and canine HF, which may underlie the increased incidence of AF in HF patients [8]. Although there is a consensus that SK channels are upregulated in HF ventricles, additional mechanistic understandings into the upregulation and heterogeneity of SK channel expression [11] will provide important insights into the clinical implications of SK channel remodeling in HF.

### Diabetes

Diabetes is a strong risk factor for both AF and HF [33, 102]. A recent study found that streptozotocin-induced diabetes in a mouse model downregulates SK2 and SK3 channels in atrial tissues by 85% and 92%, respectively, while SK1 remains the same as control [102]. Indeed, in vitro findings with HL-1 cells treated with high glucose demonstrate similar changes in SK channel expression. SK channel-associated electrical remodeling may therefore contribute to pathogenesis or increase susceptibility to AF. The underlying mechanisms for the remodeling require further investigations.

### Human calmodulinopathy

**Fig. 4:**
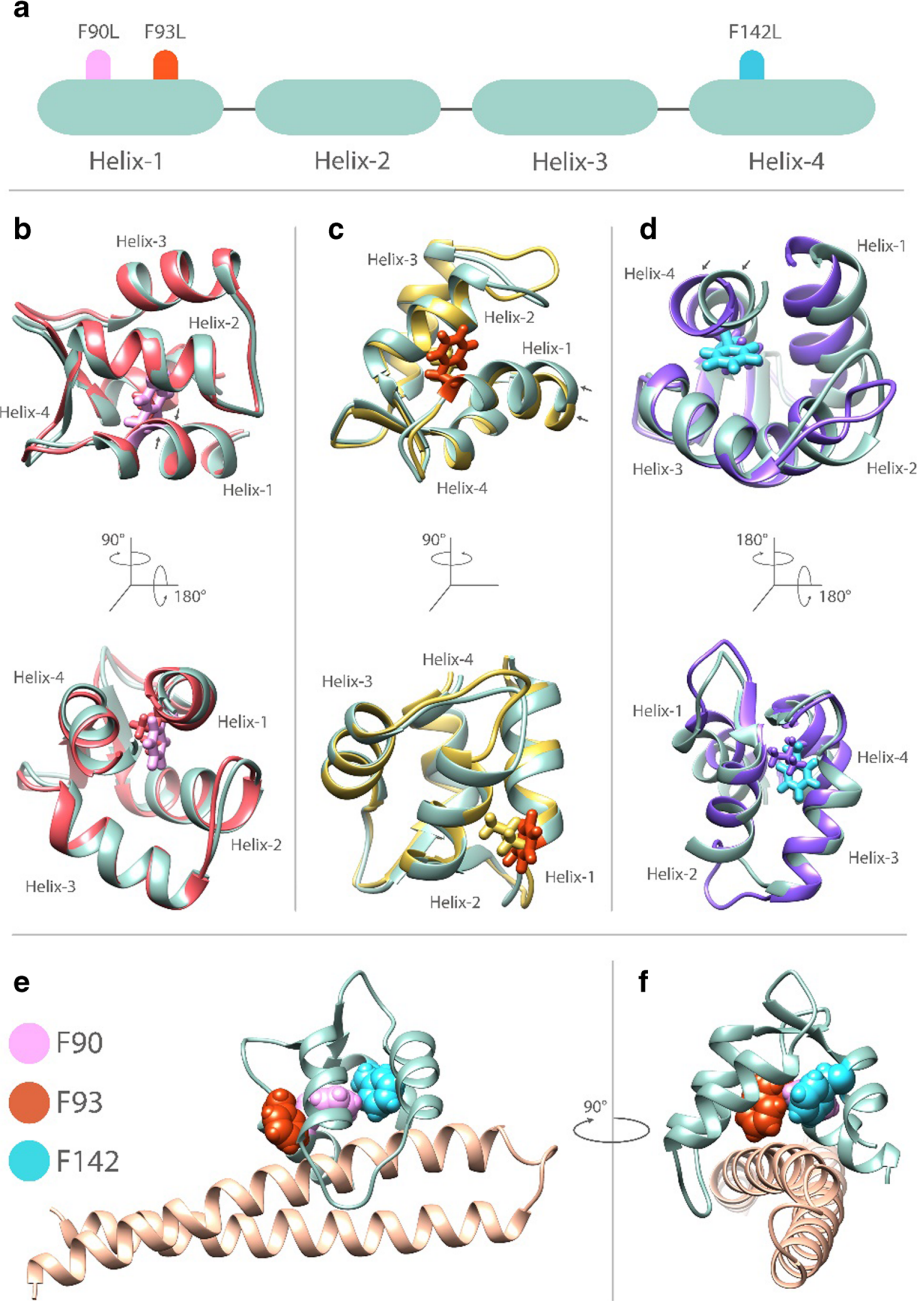
Structural modeling of the interactions between CaM mutants and SK2 CaMBD [38]. **a** Schematic of the 4 α-helices within the C-lobe of CaM. Location of mutations are shown by labels and colored markers. **b–d** Comparisons of CaM_WT_ (green) and CaM_F90L_ (red, **b**), CaM_F93L_ (yellow, **c**), and CaM_F142L_ (purple, **d**). Side chains of key amino acid residues are shown in stick representation using color scheme shown in panel **a**. Conformational changes due to CaM mutation are indicated by black arrows in each panel. **e** C-lobe of apo-CaM_WT_ (colored in green) bound to the C-terminus of *h*SK2 channel (colored in light brown). Side chains of key amino acids are shown using space-filling representation. **f** Panel E rotated 90° to the left around the Y-axis. Molecular modeling was performed in Ca^2+^ free conditions

Since SK channel activation and membrane localization are critically dependent on CaM binding to the CaMBD in the C terminus of the channel, CaM mutations, associated with human arrhythmia syndrome, significantly alter SK channel function [38, 81, 104]. Five human CaM mutations (N54I, N98S, D96V, D130G, and F90L) significantly reduces SK2 current relative to wild-type CaM without a change in the membrane or intracellular protein expression [104]. Similarly, long QT syndrome (LQTS)-associated CaM variants (D96V, D130G, F142L) and catecholaminergic polymorphic ventricular tachycardia (CPVT)-associated variant (N54I) reduces SK3 current [81]. To address the mechanistic underpinnings of the CaM mutations on SK channel function, we took advantage of human induced pluripotent stem cell-derived cardiomyocyte-like cells (hiPSC-CMs) as well as structural modeling and molecular dynamics simulation [38]. We demonstrate that human calmodulinopathy-associated CaM mutations disrupt cardiac SK channel function via distinct mechanisms. CaM_D96V_ and CaM_D130G_ mutants reduce SK currents through a dominant-negative fashion. By contrast, specific mutations replacing phenylalanine with leucine result in conformational changes that affect helix packing in the C- lobe, which disengage the interactions between apo-CaM and the CaM binding domain of SK channels (Fig. 4). Even though SK currents play a relatively minor role in normal ventricles, the channels are expressed in cardiac Purkinje cells [79], the probable site of origin of cardiac arrhythmias, including in patients with heritable arrhythmia syndrome [36, 96]. Additionally, SK channels are expressed and play important roles in pacemaking cells, including sinoatrial and atrioventricular nodes [92, 105]. Distinct mutant CaMs may significantly reduce the activation of the SK channels, decreasing the key Ca^2+^-dependent repolarization currents these channels mediate, exacerbating the effects of CaM mutations in human arrhythmia syndrome.

## SK channels in stem cell cardiomyogenesis

Activation of SK channels and maintaining the activated state by small molecule, 1-ethyl-2- benzimidazolinone (EBIO), has been reported to drive cardiomyogenesis in both the murine and the human pluripotent stem cells (PSCs) [46, 52, 67]. In the murine PSCs, SK channel activation on day 5 of the differentiated embryoid bodies improves the cardiomyogenesis efficiency, as assessed by flow cytometry with a ~4-fold increase in the fraction of troponin-positive cells [46]. Additionally, EBIO induced an upregulation of the pacemaking-specific genes (Hcn4, Tbx3, Shox2, Cx30.2, and Cx45), a downregulation of the ventricular-specific genes (Myl2v, Cx43), and an increase in the number of cardiomyocytes exhibiting pacemaker-like action potentials, suggesting that the pacemaking-like cardiomyocyte subtype was promoted over that of the ventricular-like cells. The promotion of pacemaking-like cardiomyocytes is mediated through the activation of the ERK1/2 signaling pathway [46]. EBIO-induction of cardiomyogenesis with a preferential yield for the pacemaking-like subtype was similarly demonstrated in human PSCs; however, the pacemaking-like electrophysiology was not demonstrated in the study [67]. The overexpression of the dominant SK channel isoform present in the murine PSCs, the SK4 isoform, promotes cardiomyogenesis in an inducible SK4 murine PSC line but without an apparent bias for a particular cardiomyocyte subtype [52]. Since SK4 is not the dominant SK channel isoform in human PSCs [67], SK4 is not likely the key player in EBIO-induced cardiomyogenesis in human cells like the murine PSCs.

Interestingly, a recent publication on cardiomyogenesis reported that the observed EBIO-induced increase in the cardiomyocyte yield and concurrent increase in the fraction of pacemaking-like cardiomyocytes from the human hPSCs were not attributed to directed differentiation but rather an enrichment due to a higher tolerance or survivability of cardiomyocytes—specifically those that have APs with a short APD—to EBIO-induced toxicity than the non-cardiomyocytes [42]. Indeed, the overall cell count in the EBIO-treated hPSCs was 2.5 times fewer than the control cells. The mechanism of action by EBIO was also determined to be SK4-independent since the SK4-specific activator, NS309, did not induce the same cardiomyocyte yield as that of EBIO. Only a SK2/3-selective activator, CyPPA, was able to induce similar EBIO-mediated cardiomyocyte enrichment. This is in agreement with SK2 being the dominant SK isoform in human PSCs [67]. Nevertheless, considering that none of the SK blockers tested (clotrimazole and apamin) were able to negate the EBIO-mediated cardiomyocyte enrichment, the exact enrichment mechanism by EBIO remains to be elucidated.

## SK channels as therapeutic targets

The differential expression of cardiac SK channels in autorhythmic and contractile myocytes and their heterogeneous distribution within each tissue type [15] create a remarkable and exciting opportunity for cardiac regional-specific therapy. Indeed, a major limitation of current antiarrhythmic drugs is the risk for proarrhythmia, which can be life-threatening [70]. SK channels may serve as a new therapeutic target for atrial arrhythmias. Moreover, SK channel trafficking is tightly regulated by intracellular Ca^2+^ and may serve as one future therapeutic avenue for SK channel targeting and cardiomyocyte excitability. However, SK channels are known to be upregulated in HF and may serve to increase cardiac repolarization reserve by counterbalancing the downregulation of other K^+^ channels. Therefore, elucidating SK channels’ intricate mechanisms in pathological conditions will have profound clinical implications.

## Conclusions and perspectives

The identification of cardiac SK channels opens a new avenue of research and provides therapeutic opportunities in cardiac arrhythmias. The significance of cardiac SK channels is supported by studies from multiple investigators over the past two decades. The cumulative findings so far have greatly enhanced our understanding of SK channels in the regulation of cardiac excitability and function. However, there exist many challenges and knowledge gaps. The role of SK channels in AF and ventricular arrhythmias is not entirely understood. Functional roles of SK channels in pacemaking cells and cardiac conduction systems are only beginning to be realized. The study of SK channels in intracellular organelles, including cardiac mitochondria, is in its early stage. Additionally, the local control and feedback of Ca^2+^ and SK channels within the microdomains are not well understood. On the translational level, the use of SK channel blockers in the treatment of cardiac arrhythmia and the possible pro-arrhythmic effects of SK channel blockers require further consideration and investigation.
